# Reconsidering Routine Histopathological Examination of Gallbladder Specimens in Cholecystectomy: Optimizing Clinical Practice and Resource Management

**DOI:** 10.7759/cureus.64762

**Published:** 2024-07-17

**Authors:** Zohaib Jamal, Zirong Yu, Nowera Zafar, Damon Li

**Affiliations:** 1 Department of Surgery, Redland Hospital, Bayside Health Service, Redland, AUS

**Keywords:** cancer, high-risk, gall bladder, cholecystectomy, histopathology

## Abstract

Introduction

Cholecystectomy, the surgical removal of the gallbladder, is a common procedure worldwide. Despite no visible anomalies, routine histopathological examination (HPE) of gallbladder specimens post-surgery is standard practice to exclude pathologies, notably gallbladder cancer (GBC). Incidence rates of GBC vary geographically and ethnically. Surgical intervention is recommended for advanced GBC stages, while early stages may require cholecystectomy alone. Although rare, GBC and bile duct cancers pose increased risks in certain demographics, such as women and individuals over 65. Routine HPE practices vary globally based on resource availability and GBC incidence. This study assesses the necessity of routine HPE by evaluating the selective processing of gallbladder specimens suspected of GBC, prioritizing patient safety.

Materials and methods

This retrospective cohort study conducted at Redland Hospital, a district general hospital in Australia, investigated the necessity of routine HPE for excised gallbladder specimens. Adhering to routine HPE policy, the study encompassed all elective and emergency cholecystectomies performed from January 2023 to December 2023, excluding pediatric cases, concurrent surgical procedures, and those with suspected malignancy. Demographic data, surgery indications, intraoperative findings, histopathological results, and incidental gallbladder cancer (IGC) outcomes were analyzed. Pathology reports and case documentation were reviewed for cancerous pathology indicators.

Results

Over the one-year study period from January 2023 to December 2023, a total of 266 gallbladder specimens were subjected to HPE post-cholecystectomy. Of these, 201 were female and 65 were male, yielding a male-to-female ratio of 3:1. Elective cholecystectomy was performed on 56.4% (150) of patients, while 43.6% (116) underwent emergency procedures. Laparoscopic cholecystectomy (LC) was the primary surgical approach, except for one case requiring conversion to an open procedure. None of the patients exhibited GBC; however, 3.3% (9) displayed premalignant histopathological features in their specimens.

Conclusion

In conclusion, adopting a selective approach, where only gallbladder specimens with macroscopic abnormalities undergo HPE, seems prudent, especially in regions with low GBC incidence. Our study, which revealed no cases of GBC, supports this approach. It not only reduces the risk of missing incidental carcinoma in clinically unsuspected cases but also proves cost-effective and reduces the histopathology department workload without compromising patient outcomes. Therefore, we advocate for routine macroscopic examination of gallbladder specimens for abnormalities before HPE submission, particularly in cholecystectomy patients with gallstone disease.

## Introduction

Cholecystectomy, which is the removal of the gall bladder, is one of the most commonly performed abdominal surgeries throughout the world [1]. Regardless of any readily apparent anomalies, it is a widespread practice to submit all gallbladder specimens for routine histopathological examination (HPE) after surgery [2,3]. The primary goal of the HPE is to exclude the presence of any other pathologies, particularly gallbladder cancer (GBC), which is an uncommon but most prevalent biliary system malignancy with a poor prognosis and a median survival of barely three months and a five-year survival rate of less than 5% [3,4]. The incidence of GBC ranges from approximately 0.2% to 2.9% of all cholecystectomies carried out for gall bladder-related disease, and the occurrence of GBC significantly varies among different geographical locations and ethnicities, with a difference in incidence in different regions of a country [5]. The highest incidence of GBC has been observed in North America and Northern Europe, but it is rather uncommon in East Asia, South America, and Eastern Europe [1,6]. Cholecystectomy alone is an effective treatment for patients with an accidental GBC identified at stages Tis and T1a. Surgical intervention is recommended for patients whose stage is T1b or higher [3,7].

According to the data published by the Australian Institute of Health and Welfare in 2019, GBC and bile duct cancers are rare in the Australian population, with an incidence of about three cases per 100,000 people, with an increased risk in women and those aged greater than 65 [8]. Similarly, the data published by Cancer Council Australia estimates that approximately 300 people were diagnosed with GBC in 2023, with an average age of 74 years at diagnosis [9]. The practice of routine versus selective HPE of gallbladder specimens varies widely in different parts of the world depending upon the resources and incidence of the GBC [10]. In our hospital, it is a routine practice to send all the GB specimens after cholecystectomy for routine HPE.

The aim of the study was to investigate the role of routine HPE of gall bladder specimens after a routine cholecystectomy and adopt a policy of processing only the gall bladder specimen with clinical and intraoperative suspicion of GBC without compromising patient safety.

## Materials and methods

This retrospective cohort study was carried out at Redland Hospital, Metro South Health, which is a district general hospital in Cleveland, Queensland, Australia. In our hospital, all excised gallbladder specimens undergo routine HPE as part of our standard protocol. The project was submitted to the Metro South Human Research Ethics Committee, which is our institutional Ethics Review Committee (ERC), and was deemed exempt from the full ethical review, reference number EX/2024/QMS/106088. 

Patients between the ages of 16 to 84 years of age who underwent elective or emergency cholecystectomies performed over a year from January 2023 to December 2023 at Redland Hospital, encompassing both laparoscopic and open procedures, were included in the study. The exclusion criteria consisted of pediatric patients, cases in which gallbladders were removed during other concurrent surgical procedures, and those with preoperative diagnoses or strong suspicion of malignancy.

The data was collected retrospectively from the integrated electronic Medical Record (ieMR) software in use at the Redland hospital, with patient information retrieved using their unique record numbers (URN). The surgical operation notes were also studied using the ieMR power charts. The results of the scans, including the images and the reports, were acquired using the Picture Archiving and Communication System (PACS) used across Queensland. Data concerning demographic parameters, indications for surgery, intraoperative findings, histopathological results, and clinical outcomes related to incidental gallbladder cancer (IGC) were meticulously collected and analyzed. Furthermore, clinical notes and pathology reports were also reviewed to identify any intraoperative or macroscopic abnormalities indicative of cancerous pathology. The criteria for identifying macroscopic abnormalities included unusual wall thickening, masses or nodules, mucosal irregularities, abnormal shape, perforation or necrosis, and unusual pigmentation or calcification. A predesigned proforma was utilized to collect the information, and a spreadsheet was created to record the gathered data using the Microsoft Excel software (Microsoft, Redmond, US). Statistical analyses were performed utilizing the Statistical Package for the Social Sciences (SPSS) software version 28.0 (SPSS Inc., Chicago, IL, USA). The data was analyzed using the chi-square test and a significance level of 0.05 was utilized.

## Results

**Figure 1:**
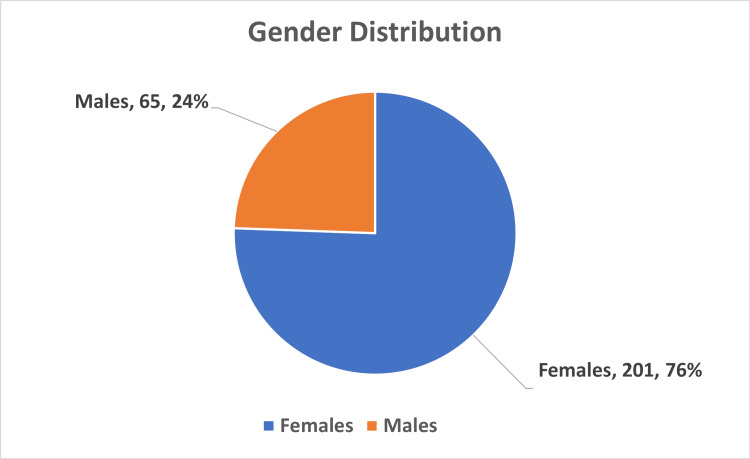
Gender distribution of the study population

During the study period of one year, from January 2023 to December 2023, a total of 266 gallbladder specimens submitted for HPE after cholecystectomy were analyzed. There were 201 females and 65 males, and the male/female ratio was 3:1 (Figure 1). The age ranged from 18 to 84 years, with a mean age of 49 years, as shown in Table 1. 

**Table 1:** Age distribution of the study population

Age of patients (years)	No. of patients (n=266)	Percentage (%)
18-30	43	16.1
31-40	47	17.6
41-50	51	19.1
51-60	52	19.5
61-70	44	16.5
71-80	27	10.1
81-84	02	0.75

**Figure 2:**
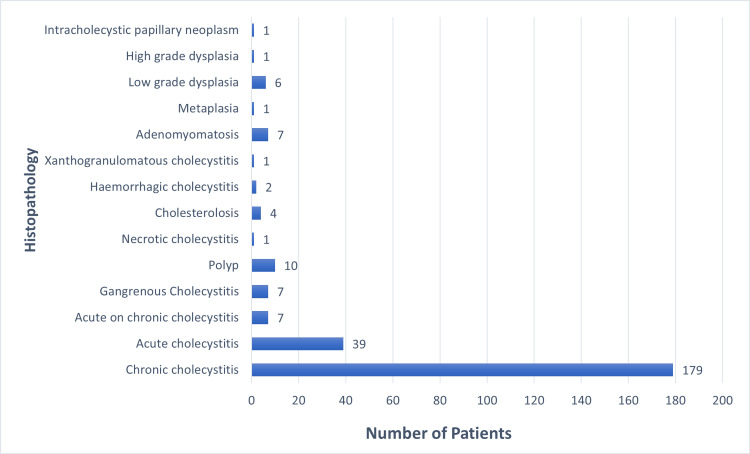
Details of histopathology findings from 266 cholecystectomy specimens

Within the cohort of 266 patients, 150 individuals (56.4%) underwent elective cholecystectomy, while 116 patients (43.6%) necessitated emergency procedures. Laparoscopic cholecystectomy (LC) was performed on all patients except for one instance where conversion to an open procedure was required. Notably, none of the patients in our study presented with GBC. However, a total of nine patients (3.3%) exhibited premalignant histopathological features in their gallbladder specimens. Figure 2 shows a comprehensive illustration of the histopathological findings obtained from the 266 cholecystectomy specimens analyzed in this study.

## Discussion

Routine versus selective HPE of the gall bladder specimen after a cholecystectomy has been controversial in the literature. The debate surrounding selective HPE of gallbladder specimens hinges on three key arguments. Proponents assert that routine HPE is unnecessary due to the low likelihood of encountering IGC in visually normal specimens, particularly at early stages [11-13]. Additionally, they argue that early-stage GBCs, such as those classified as Tis and T1a, often appear grossly normal and thus do not require further treatment beyond simple cholecystectomy, while also highlighting the burden imposed on histopathology departments in terms of their workload and hospital funding and resources by routine examination of all specimens [14-16]. On the contrary, opponents argue that GBC poses significant mortality risks, emphasizing the importance of early detection of incidental malignancies to ensure favorable patient outcomes.

Overall, the incidence of GBC varies between 0.2% and 2.9%, depending on the geographical location and ethnicity [5]. Interestingly, the occurrence of incidentally discovering carcinoma in gallbladder specimens without clinical suspicion is exceedingly rare, as documented by various studies. Bazoa et al. (2007) reported a frequency of 0.1%, while Bawahab et al. (2013) noted 0.37%, and Mittel et al. (2010) found it to be 0.99% [11,17,18]. Our study did not show any evidence of GBC in any of the specimens sent for HPE, in keeping with the data published by the Australian Institute of Health and Welfare in 2019 and Cancer Council Australia, thus indicating GBC to be a rare cancer in the Australian population [8,9]. Similarly, van Vliet et al. elucidated that out of the 1375 gallbladder specimens subjected to HPE, none exhibited incidental GBC [19]. Investigations carried out by Oguzhan et al. and Davide et al. underscore that the prevalence of incidental GBC in studies conducted within regions characterized by low incidences of the disease is anticipated to be minimal [20,21].

Conversely, research conducted in regions with elevated risk factors, such as India and Pakistan, documents increased incidences of GBC. In their systematic literature review comprising data from 21 studies, Jamal et al. discerned the Asian population exhibits an elevated risk for GBC, presenting a higher reported incidence in contrast to the lower-risk European population [22]. The authors observed that during the intra-operative examination, macroscopic abnormalities were evident in 92% of GBCs and, therefore, propose that specimens appearing macroscopically normal, particularly in low-risk patients (i.e., Europeans under 60 years of age), may not necessitate formal HPE [22]. Yadav et al. conducted research within the North Indian population, revealing an incidence rate of 1.26% [23]. Similarly, Siddiqui et al. observed a prevalence of 2.8% for GBC within the Pakistani population [10]. The variability in gallbladder malignancy rates across different geographical locations and ethnicities underscores the potential necessity for adjusting clinical practices based on patient demographics.

In our study, the predominant diagnosis upon HPE of gallbladder specimens was chronic cholecystitis, consistent with findings reported by Faten et al. [24]. Nonetheless, contrasting findings were reported by Oguzhan et al. and Davide et al., who documented notably high incidences of chronic cholecystitis in their respective studies, with rates of 92.3% and 99.3%, respectively [20,21]. The occurrence of premalignant lesions was observed to be 3.3%, contrasting with the rates of 0.1% and 0.31% documented by Aboutaleb et al. and Benkhadoura et al., respectively [25]. The particulars of patients exhibiting premalignant lesions have been consolidated in Table 2.

**Table 2:** Details of patients with a histopathological diagnosis of metaplasia, high-grade dysplasia, low-grade dysplasia, and ICPN USS: ultrasound scan; CT: computed tomography; MRCP: magnetic resonance cholangiopancreatography; IOC: intraoperative cholangiogram; CBD: common bile duct; IPMNs: intraductal papillary mucinous neoplasms; ICPN: intracholecystic papillary neoplasm

Patient	Age	Gender	Indication for surgery	Preoperative/imaging findings	Intraoperative finding	Histopathology
1.	68	M	Biliary colic	USS: Multiple gallstones present, uncomplicated, along with mild hepatic steatosis. CT: Normal.	A thin-walled gallbladder with an adequate-length cystic duct that is quite dilated. The intrahepatic anatomy is complete, with no filling defects observed. The cystic duct exhibits a good taper, and there is good flow through to the second part of the duodenum.	Metaplasia
2.	55	M	Gallstone pancreatitis	CT: Suggestive of a pancreatic lesion. MRCP: No pancreatic lesion detected.	Omental adhesions to the anterior abdominal wall/mesh, thickened gallbladder wall, intrahepatic body, wide cystic duct, clear bile expressed from the cystic duct/common bile duct, fatty liver, and the anterior branch of the cystic artery crossing the cystic duct/Hartmann’s pouch.	Low-grade dysplasia
3.	57	F	Biliary colic	USS: Cholelithiasis observed with no features indicative of acute cholecystitis. No evidence of bile duct dilatation or obvious choledocholithiasis.	Mildly inflamed gallbladder and omental adhesions consistent with chronic, recurrent cholecystitis are noted. Additionally, a small umbilical hernia repaired with sutures is observed. IOC reveals normal results, with no filling defects observed. There is distal tapering and appropriate flow into the second part of the duodenum. The intrahepatic ducts appear normal.	Low-grade dysplasia
4.	39	F	Biliary colic	USS: Cholelithiasis noted, comprising one large stone measuring 2 cm in size.	The gallbladder appears normal, with a single stone located in the gallbladder neck. IOC shows fast flow into the second part of the duodenum, with no filling defects observed. The intrahepatic duct anatomy appears normal.	Low-grade dysplasia
5.	70	M	Biliary colic	USS: Multiple stones observed; CBD appears normal.	A fatty infiltrative liver and omental and duodenal adhesions to the gallbladder wall, consistent with recurrent biliary colic. IOC revealed normal anatomy, tapering of the CBD, and no filling defects. There was appropriate flow to the second part of the duodenum, with visualization of the anterior and posterior branches of the hepatic branch. Additionally, iatrogenic perforation of the gallbladder occurred with stone spillage, necessitating retrieval of stones with suction and a grasper.	Low-grade dysplasia
6.	55	F	Biliary colic	USS: Cholelithiasis detected, featuring a mobile 16 mm calculus. No signs of cholecystitis are evident. Additionally, mild hepatic steatosis is observed.	A distended thin-walled gallbladder with no adhesions, along with a thin cystic duct. Notably, there is a very large posterior cystic artery and a small anterior cystic artery. IOC revealed normal results, with no filling defect observed in the CBD. Additionally, there was normal tapering of the distal CBD, and the right and left hepatic branches appeared normal. Contrast flow into the second part of the duodenum was observed.	Low-grade dysplasia
7.	72	F	Biliary colic	USS: Cholelithiasis noted, with multiple stones measuring up to 14 mm. CBD size appears normal.	A thin-walled gallbladder was observed. During diathermy of the gallbladder off the cystic plate, two small gallstones were inadvertently dropped intraabdominally but were subsequently retrieved. IOC revealed contrast flow through to the second part of the duodenum with no filling defects. Additionally, a dilated CBD was noted with stricturing, which appears appropriate for the patient's age. Normal intrahepatic ductal anatomy was also demonstrated.	Low-grade dysplasia
8.	84	F	Acute cholecystitis	USS: Calculus cholecystitis diagnosed. CBD appeared slightly prominent; no obvious obstructive lesion identified. MRCP: Presence of a gallbladder calculus with associated gallbladder wall and subserosal edema noted. Suspicion of a tiny common bile duct calculus. Prominent biliary tree observed. Additionally, several IPMNs identified in the pancreas, along with a diverticulum in the descending duodenum.	The gallbladder appeared mildly edematous with adhering omentum. IOC revealed a dilated CBD with a distal filling defect and a small amount of contrast passing to the duodenum, along with good opacification of intrahepatic ducts. Upon completion of the IOC, no obvious distal filling defect was observed, although confidence in the clearance of CBD stones was not absolute.	High-grade dysplasia
9.	42	M	Gall bladder polyp	USS: 11 mm solitary non-mobile echogenic structure detected along the anti-dependent gallbladder wall with a broad base but no focal underlying gallbladder wall thickening, likely indicating a gallbladder polyp. CT: Normal.	The gallbladder appeared thin-walled, with no evidence of polyps or other abnormalities on the serosal surface. The liver was normal, and there was no evidence of any peritoneal deposits. IOC showed an adequate length cystic duct, complete intrahepatic anatomy, and no filling defects. Additionally, there was good taper and flow through to the second part of the duodenum.	ICPN

## Conclusions

In conclusion, the adoption of a selective approach, wherein only gallbladder specimens displaying macroscopic abnormalities undergo HPE, appears to be a prudent strategy, particularly in regions with a minimal incidence of GBC. Our findings, which revealed a zero incidence of GBC, align with this recommendation. This approach not only mitigates the risk of overlooking incidental carcinoma in clinically unsuspected cases but also proves to be cost-effective and reduces the workload on histopathology departments without compromising patient outcomes. Hence, we advocate for the routine macroscopic examination of gallbladder specimens for abnormalities before deciding on submission for HPE, especially in patients undergoing cholecystectomy for gallstone disease. The study's strengths lie in its comprehensive analysis of a large cohort of gallbladder specimens with standardized HPE protocols, providing clinically relevant insights into post-cholecystectomy pathology practices. However, limitations include its retrospective single-center design, potential biases from exclusion criteria, and the absence of advanced diagnostic techniques, warranting cautious interpretation of findings and suggesting avenues for future research.
